# In-Depth Analysis of the Essential Oil of *Achillea erba-rotta* subsp. *moschata* (Asteraceae)

**DOI:** 10.3390/plants15203176

**Published:** 2026-10-16

**Authors:** Aldo Tava, Elisa Biazzi, Pietro Fusani

**Affiliations:** 1 Consiglio per la ricerca in agricoltura e l’analisi dell’economia agraria (CREA), Centro di ricerca Zootecnia e Acquacultura, Viale Piacenza 29, 26900 Lodi, Italy; elisa.biazzi@crea.gov.it; 2 Consiglio per la ricerca in agricoltura e l’analisi dell’economia agraria (CREA), Centro di ricerca Foreste e Legno, Piazza Nicolini 6, 38100 Trento, Italy; pietro.fusani@crea.gov.it

**Keywords:** essential oil composition, variability, camphor, thujones, caryophyllene derivatives

## Abstract

*Achillea erba-rotta* subsp. *moschata* (Wulfen) Vacc. (Sin.: *A. moschata* Wulfen) is a plant belonging to the Asteraceae family and is traditionally used for its medicinal and aromatic properties. Nowadays, it is primarily used as an ingredient of several commercial liqueurs. The essential oil from a representative number of *A. erba-rotta* subsp. *moschata* plants collected from the Eastern Italian Alps and cultivated in the same experimental conditions were investigated by GC/FID and GC/MS methods. A great variability among analyzed plant samples was recorded for the essential oil qualitative and quantitative composition. Oxygenated monoterpenes were the main class of detected compounds, of which *cis*- and *trans*-thujones (from nil to 85.5% of the total oil), camphor (from 0.2 to 55.6%) and 1,8-cineol (from 0.3 to 19.7%) were the most abundant. Among sesquiterpenes, the group of caryophyllenes, including caryophyllene and its oxygenate derivatives, were quoted from 3.3 to 29.9%, while *β*-eudesmol comprised from nil to 16.9% of the total oil. Given the known toxicity of thujones for human consumption, the results of this study are of particular importance for selecting plants with lower thujone content in the framework of a possible breeding program.

## 1. Introduction

*Achillea erba-rotta* subsp. *moschata* (Wulfen) Vacc., Syn. *A. moschata* Wulfen (Asteraceae), is a perennial herb endemic to the central and eastern Alps, growing on siliceous cliffs, screes and moraines at altitudes above 1800 m a.s.l. Its deeply pinnatifid leaves allow distinguishing it from *A. erba-rotta* subsp. *erba-rotta*, characterized by a simple leaf, dentate at the apex, and distributed in the western Alps [1,2,3].

*A. erba-rotta* subsp. *moschata* has been traditionally used both as a medicinal, for its digestive, antispasmodic and carminative properties, and as an aromatic plant, to aromatize distillates and liquors, mainly in the Alpine area [4,5]. The specific name of this taxon, *moschata*, is used indeed to designate different plant species characterized by a strong, aromatic smell [6]. Nowadays, the species is mostly used for its aromatic features, to produce liquors and bitters, mainly in the Alpine regions of Italy, where an annual demand by the processing industry of 8 t of dry raw material has been estimated [7,8].

The aromatic properties of the flowering aerial parts of *A. erba-rotta* subsp. *moschata* are evidently due to their volatile composition, which has been the subject of few studies to date. Maffei et al. [9], in a study on the characterization of the essential oil extracted from different species of the genus *Achillea* L., reported 39 components in the volatile fraction of *A. erba-rotta* subsp. *moschata*, of which 27 were monoterpenes, nine sesquiterpenes, and three phenols. Vitalini et al. [10] and later Tava et al. [11] reported 40 volatile compounds, 28 of which were monoterpenes and 12 sesquiterpenes. Vitalini et al. [12] successively reported 20 volatile compounds, three of which were sesquiterpenes. In a study focused on the characterization of different natural populations of the species, we detected 45 compounds, 32 of which were monoterpenes and 13 sesquiterpenes [13]. In a successive study aimed at comparing different cultivated accessions from the morphological, agronomic, and chemical perspectives, we identified 40 compounds of the volatile fraction, 27 of which were monoterpenes and 13 sesquiterpenes [14]. It is worth noting that the above-mentioned authors were able to identify all the monoterpenes detected, while most of the sesquiterpenes remained unidentified [9,10,11,12,13,14]. This fact, together with the wide variability observed in the volatile composition of the species, has suggested the opportunity to deepen the characterization of the volatile fraction already reported by the same authors [14]. Thus, the aim of this study was to improve the knowledge on the chemical composition of the essential oil of *A. erba-rotta* subsp. *moschata*, especially regarding the class of sesquiterpenes, and to investigate the variability of the composition of the volatile fraction in this species.

## 2. Results and Discussion

The essential oil yield of the twenty *A. erba-rotta* subsp. *moschata* samples considered in this study ranged from 0.28 to 0.61% dry matter (see Table 1), in agreement with data reported in the literature [11,13,14]. The unfractionated essential oils were analyzed as such by GC/FID and GC/MS (Table 2). Additionally, a portion of representative essential oil, obtained by mixing equal amounts of each of the obtained oils, was successively separated into three fractions by column chromatography, eluting with solvents with increasing polarity (see experiments below). Fractionation of the essential oil was performed to obtain more detailed information on the chemical structures of its individual constituents. Chromatographic separation did not reveal any minor compounds that co-eluted with, or were obscured by, major peaks in the unfractionated essential oil. Combined with mass spectral interpretation, this approach enabled a more accurate characterization and identification of the compounds present in the essential oil. The use of solvents with increasing polarity allowed the compounds to be separated according to their polarity. Thus, hydrocarbons were eluted with pentane and collected in fraction N, whereas less polar oxygenated compounds, such as ketones and esters, were eluted using pentane containing 5% diethyl ether and collected in fraction P1. Finally, more polar compounds, such as alcohols, were recovered using diethyl ether and collected in fraction P2.

In addition to separating the compounds according to their chemical nature, in some cases this fractionation allowed the enrichment of fractions containing compounds that were present only in small amounts in the whole essential oil, thereby facilitating their identification and characterization. This approach proved to be particularly useful for the identification of several compounds, especially sesquiterpenes, including caryophyllene and its derivatives. Caryophyllene, a C_15_ hydrocarbon (compound **35**, see Table 2), was eluted in fraction N, in accordance with its non-polar nature. Its derivatives, namely the oxide **42** and the ketones **41** and **43**, were recovered in fraction P1, whereas the hydroxylated derivatives **49**, **50**, **53**, and **55** were eluted in the more polar fraction P2.

All these fractions were analyzed by GC/FID and GC/MS. Results of these investigations are reported in Table 2, in which compounds are listed according to their retention indices (AI) on a DB-5 column. An indication of the fraction in which they were detected after chromatographic separation is also reported. These analyses revealed the presence of 63 compounds, 59 of which were identified (Table 2). Among the identified compounds, 21 were detected for the first time in *A. erba-rotta* subsp. *moschata*: the oxygenated monoterpenes 3-thujanol (**23**), carvone (**29**) and *trans*-sabinyl acetate (**32**); the sesquiterpene *δ*-amorphene (**38**); the lactones 13-tetradecanolide (**51**) and 15-hexadecanolide (**63**); and finally, 14 sesquiterpenes, that is, compounds from **38** to **50**, with the exclusion of **40** and **42**, already reported in [14], and those from **53** to **62**, with the exclusion of chamazulene (**61**), all listed in Table 2.

A great variability in the qualitative and quantitative composition of the essential oil from the twenty samples of *A. erba-rotta* subsp. *moschata* was found. As a general trend, oxygenated monoterpenes were detected as the most abundant class of compounds, ranging from 43.2% to 90.4% of the total oils, followed by oxygenated sesquiterpenes (5.2–45.6%), monoterpene hydrocarbons (0.3–13.6%) and sesquiterpene hydrocarbons (0.6–4.9%). These differences are also evident when individual compounds are considered. Although detected in variable amounts, camphor (**21**, 0.2–55.6%), cis-thujone (**16**, 0–71.2%) and trans-thujone (**17**, 0–64.5%) were confirmed to be the most representative compounds of the essential oil of this species, together with 1,8-cineole (**11**, 0.3–19.7%) and terpinen-4-ol (25, 0.9–5.5%), as previously reported [9,10,11,12,13,14]. Among sesquiterpenes, *β*-eudesmol (**52**, 0–16.9%), *E*-nerolidol (**40**, 0–6.7%) and sesquicineole (**37**, 0–4.8%) were quoted in variable amounts with higher relative percentage composition in some of the examined *A. erba-rotta* subsp. *moschata* samples (Table 2). Another characteristic of these oils, indeed, is the presence of a relatively higher content of caryophyllene and a series of its oxygenated derivatives (Figure 1), which are quoted all together, from 3.3 to 29.9% of the total oil. Caryophylla-2(12),6(13)-dien-5*β*-ol (**50**, 0.7–10.9%) and caryophyllene oxide (**42**, 0.7–7.7%) are the most representative oxygenated caryophyllene derivatives of this series. Caryophyllene and its oxide are found in several volatile oils; however, the presence of the other caryophyllene oxygenated derivatives (ketones and alcohols) is not so common, although not unusual for the genus *Achillea* L., as these compounds have been previously identified, although in lesser amounts, in the volatile oil from several species belonging to this genus [15,16,17,18,19,20,21,22]. If compared with the literature data, the presence of a relatively higher content of these compounds in the essential oil of *A. erba-rotta* subsp. *moschata* seems to be a characteristic feature of this species. It is worth noting that, among the identified components of the essential oil, 13-tetradecanolide (**51**) and 15-hexadecanolide (**63**) were detected, although at low relative percentages (0. 2 and 0.6% on average, respectively).

**Table 2:** Essential oil composition (%) of the twenty investigated samples of *A. erba-rotta* subsp. *moschata* flowering tops.

#	Compound ^a^	Fraction Detected ^b^	AI Tab ^c^	AI ^d^	Analyzed Samples																				Min	Max	Mean	SD
					1	2	3	4	5	6	7	8	9	10	11	12	13	14	15	16	17	18	19	20				
**1**	santolina triene	W, N	906	907	-	0.7	-	tr	0.8	0.3	-	-	-	0.1	-	-	-	-	0.1	0.4	-	-	-	-	0.0	0.8	0.1	0.2
**2**	*α*-thujene	W, N	924	924	0.5	0.7	0.1	0.2	0.4	0.1	0.1	0.1	0.5	0.3	0.2	tr	tr	0.1	tr	0.1	-	tr	0.1	0.1	0.0	0.7	0.2	0.2
**3**	*α*-pinene	W, N	932	929	0.6	0.5	0.4	1.0	0.6	0.5	0.1	tr	0.6	0.4	0.1	-	tr	0.1	0.3	0.5	-	tr	0.2	0.1	0.0	1.0	0.3	0.3
**4**	camphene	W, N	946	942	0.6	0.6	0.7	2.3	1.9	1.2	0.1	-	2.4	1.7	-	-	tr	0.3	1.1	1.8	-	tr	0.2	0.2	0.0	2.4	0.8	0.8
**5**	sabinene	W, N	969	969	1.0	1.6	0.7	0.8	1.1	0.4	0.5	0.2	1.2	0.8	0.4	0.1	0.3	0.8	0.5	1.7	tr	0.2	0.8	0.8	0.0	1.7	0.7	0.5
**6**	*β*-pinene	W, N	974	973	0.2	0.3	0.1	1.3	0.6	-	-	-	0.4	0.1	-	-	-	0.1	-	0.3	-	-	tr	-	0.0	1.3	0.2	0.3
**7**	*α*-phellandrene	W, N	1002	1001	tr	3.4	tr	tr	2.1	1.4	-	-	0.1	0.5	-	0.3	-	tr	0.6	3.2	-	tr	0.1	0.4	0.0	3.4	0.6	1.1
**8**	*α*-terpinene	W, N	1014	1014	0.8	0.8	0.8	0.5	0.3	0.3	0.2	0.2	0.7	0.2	0.2	0.2	0.1	0.2	0.1	0.3	-	0.1	0.2	0.4	0.0	0.8	0.3	0.3
**9**	*p*-cymene	W, N	1020	1022	1.9	2.3	2.2	3.7	1.3	1.8	0.9	0.7	1.9	1.3	1.0	0.3	0.5	1.3	1.4	2.2	0.2	0.9	1.3	1.7	0.2	3.7	1.4	0.8
**10**	limonene	W, N	1024	1025	0.4	0.4	0.2	0.2	0.3	0.2	0.1	-	0.3	0.4	0.1	0.5	0.1	0.1	0.1	0.2	-	0.1	0.1	0.2	0.0	0.5	0.2	0.1
**11**	1,8-cineole	W, P1	1026	1028	7.4	7.4	5.9	19.7	13.9	12.3	1.1	0.6	13.2	7.8	0.3	4.5	0.5	5.8	3.1	8.5	2.6	3.5	4.4	5.2	0.3	19.7	6.4	5.1
**12**	*γ*-terpinene	W, N	1054	1056	2.1	1.8	1.7	1.3	0.7	1.1	0.6	0.5	1.6	0.7	0.5	0.2	0.3	0.7	0.5	1.1	0.1	0.4	0.8	1.2	0.1	2.1	0.9	0.6
**13**	*cis*-sabinene hydrate	W, P2	1065	1065	3.3	2.2	2.9	1.1	0.7	2.0	0.6	0.9	3.0	1.6	0.5	1.6	0.3	0.9	0.5	1.6	0.7	1.0	1.6	3.7	0.3	3.7	1.5	1.0
**14**	terpinolene	W, N	1086	1086	0.4	0.4	0.4	0.2	-	-	-	-	0.3	0.2	0.1	tr	0.1	0.1	0.1	0.2	-	0.1	0.1	0.4	0.0	0.4	0.1	0.1
**15**	*trans*-sabinene hydrate	W, P2	1098	1097	1.0	0.8	0.9	0.7	0.5	0.9	0.3	0.4	0.2	0.6	0.3	1.1	0.2	0.6	0.3	0.7	0.6	tr	0.8	1.3	0.0	1.3	0.6	0.3
**16**	*cis*-thujone	W, P1, P2	1101	1104	22.5	14.5	3.9	12.9	0.3	0.3	15.9	23.3	0.8	1.8	71.2	2.8	51.3	45.4	-	tr	2.1	26.3	32.1	18.6	0.0	71.2	17.3	20.0
**17**	*trans*-thujone	W, P1, P2	1112	1115	3.1	2.7	39.5	2.6	0.5	1.9	64.5	61.0	0.2	0.3	6.6	0.2	34.2	15.7	-	-	-	4.3	4.6	3.3	0.0	64.5	12.3	20.5
**18**	chrysantenone	W, P1	1124	1120	0.7	0.1	1.1	0.4	0.1	0.7	0.8	1.1	0.5	0.5	0.4	6.5	-	0.6	0.3	0.7	0.7	0.4	0.5	0.9	0.0	6.5	0.9	1.3
**19**	*cis*-sabinol	W, P2	1131	1131	0.2	3.2	0.1	0.3	2.6	2.5	0.3	0.2	0.2	0.7	0.1	0.2	0.1	0.1	1.4	3.4	3.9	0.5	0.2	0.9	0.1	3.9	1.0	1.3
**20**	*trans*-sabinol	W, P2	1136	1137	0.2	0.2	0.2	0.3	0.3	0.4	0.3	0.2	0.2	0.3	0.1	-	0.2	0.2	0.1	0.2	0.4	0.2	0.2	0.2	0.0	0.4	0.2	0.1
**21**	camphor	W, P1, P2	1141	1142	31.2	18.9	14.2	30.2	42.8	36.1	2.6	0.2	42.4	53.9	1.0	22.3	0.8	9.6	55.6	46.2	14.9	11.1	22.6	27.7	0.2	55.6	24.2	17.8
**22**	pinocarvone	W, P1	1160	1160	0.3	0.3	0.4	0.6	0.4	0.5	tr	tr	0.4	0.4		0.2	0.1	-	0.2	0.3	0.6	0.3	0.4	0.3	0.0	0.6	0.3	0.2
**23**	3-thujanol	W, P2	1164	1163	-	-	-	-	4.2	5.4	-	-	-	-	-	-	-	-	-	-	-	-	-	-	0.0	5.4	0.5	1.5
**24**	borneol	W, P2	1163	1163	0.2	2.1	0.3	1.1	0.7	-	0.2	0.1	2.2	2.4	0.1	0.5	-	0.2	3.7	1.9	4.1	2.3	1.0	0.9	0.0	4.1	1.2	1.2
**25**	terpinen-4-ol	W, P1, P2	1174	1176	5.0	5.5	4.6	2.7	1.9	3.6	1.2	1.6	5.2	3.2	1.4	4.8	0.9	2.0	1.0	2.3	3.2	3.6	3.1	5.3	0.9	5.5	3.1	1.5
**26**	*α*-terpineol	W, P2	1186	1190	1.3	1.1	0.6	2.0	1.9	2.3	0.1	0.2	2.0	2.0	0.1	2.9	0.1	0.7	1.2	1.7	4.3	2.9	1.2	1.7	0.1	4.3	1.5	1.1
**27**	*trans*-carveol	W, P2	1215	1213	0.2	0.2	0.2	0.1	0.1	0.1	0.1	0.2	0.1	0.1	0.1	7.6	-	0.2	0.1	0.1	-	-	0.1	0.2	0.0	7.6	0.5	1.7
**28**	*cis*-carveol	W, P2	1226	1225	0.6	0.2	-	0.1	0.1	0.1	-	-	0.1	0.1	-	0.1	-	tr	0.8	1.0	-	-	0.3	0.3	0.0	1.0	0.2	0.3
**29**	carvone	W, P1	1239	1243	0.2	0.1	tr	0.1	0.1	0.1	-	-	0.1	0.1	-	-	-	0.1	0.6	0.2	-	-	0.3	0.1	0.0	0.6	0.1	0.1
**30**	*cis*-chrysantenyl acetate	W, N, P1	1261	1260	-	-	-	-	0.2	-	-	-	-	-	-	-	-	-	-	-	-	-	0.3	0.1	0.0	0.3	0.1	0.1
**31**	bornyl acetate	W, N, P1	1287	1286	0.9	8.0	0.9	3.6	2.4	3.5	0.4	-	0.8	0.3	-	-	0.1	0.7	3.5	1.2	3.1	1.6	6.7	3.1	0.0	8.0	2.0	2.2
**32**	*trans*-sabinyl acetate	W, N, P1	1289	1292	0.9	0.4	0.1	0.1	0.1	0.1	-	-	-	-	-	-	-	-	0.1	0.1	0.3	0.2	-	-	0.0	0.9	0.1	0.2
**33**	thymol	W, P2	1289	1296	0.4	0.9	0.3	0.5	0.1	0.4	0.1	0.1	0.2	0.2	0.6	1.8	0.1	0.3	0.7	0.5	1.6	1.1	1.0	1.0	0.1	1.8	0.6	0.5
**34**	carvacrol	W, P2	1298	1305	0.2	0.2	0.4	0.2	0.1	0.1	0.5	0.5	0.1	0.1	0.3	0.2	0.3	0.3	0.3	0.2	0.3	0.3	0.2	0.3	0.1	0.5	0.3	0.1
**35**	*E*-caryophyllene	W, N	1417	1418	0.7	1.0	0.5	0.5	0.5	0.8	0.8	0.3	1.6	1.5	0.6	3.7	0.7	0.7	0.9	1.6	2.2	1.4	2.2	1.5	0.3	3.7	1.2	0.8
**36**	*α*-humulene	W, N	1452	1453	0.1	0.1	0.1	tr	0.1	0.1	0.1	tr	0.1	0.2	0.1	0.3	0.1	0.1	0.1	0.2	tr	0.1	0.2	0.2	0.0	0.3	0.1	0.1
**37**	sesquicineole	W, N, P1	1515	1515	1.4	2.9	-	-	4.8	3.4	-	-	3.4	0.3	-	0.5	-	-	-	-	0.3	0.2	-	-	0.0	4.8	0.9	1.5
**38**	*δ*-amorphene	W, N	1511	1525	tr	tr	-	0.2	-	-	0.1	0.2	-	0.2	0.3	0.9	0.1	0.2	-	0.2	-	1.2	-	-	0.0	1.2	0.2	0.3
**39**	elemol	W, P2	1548	1552	0.1	-	-	-	-	-	-	-	-	0.1	-	-	-	-	3.9	2.7	-	2.0	-	tr	0.0	3.9	0.4	1.0
**40**	*E*-nerolidol	W, P2	1561	1566	3.1	2.6	-	-	0.4	-	tr	1.0	0.6	0.8	0.9	0.1	-	0.5	6.7	0.1	-	-	1.2	2.8	0.0	6.7	1.0	1.6
**41**	caryophylla-2(12),6(13)-dien-5-one	W, P1	1559 ^e^	1577	0.8	0.9	0.9	1.1	0.6	1.4	0.9	0.6	1.5	1.5	1.3	3.6	0.7	0.9	0.5	0.9	2.5	1.9	1.3	1.6	0.5	3.6	1.3	0.7
**42**	caryophyllene oxide	W, P1	1582	1584	1.2	1.7	0.9	1.5	1.2	2.2	0.9	0.7	2.4	3.0	1.7	5.5	0.8	1.1	1.2	2.0	7.7	5.2	2.2	3.5	0.7	7.7	2.3	1.8
**43**	caryophylla-2(12),6-dien-5-one	W, P1	1581 ^e^	1602	0.1	0.1	0.3	0.1	0.1	0.2	0.1	0.1	0.2	0.3	0.2	0.6	-	-	-	-	0.5	0.3	-	-	0.0	0.6	0.2	0.2
**44**	*β*-oplopenone	W, P1	1607	1607	0.1	0.1	0.4	0.2	0.4	0.1	0.3	0.1	-	-	0.4	0.4	0.4	0.1	-	-	0.3	2.1	0.3	0.2	0.0	2.1	0.3	0.4
**45**	humulene oxide II	W, P1, P2	1608	1612	0.1	0.2	-	0.1	0.3	0.3	0.3	0.4	0.1	0.1	0.3	-	0.1	0.3	0.1	0.1	2.5	0.1	0.2	0.3	0.0	2.5	0.3	0.5
**46**	muurola-4,10(14)-dien-1*β*-ol	W, P2	1630	1632	-	-	-	-	-	-	-	-	-	-	1.2	1.6	-	-	-	-	-	-	-	-	0.0	1.6	0.1	0.4
**47**	*E*-sesquilavandulol	W, P2	1631	1633	-	-	-	0.2	-	-	0.1	0.1	-	-	-	-	-	0.1	-	-	-	2.6	-	-	0.0	2.6	0.2	0.6
**48**	*γ*-eudesmol	W, P2	1630	1634	-	-	-	-	-	-	-	-	-	-	-	-	-	-	2.9	0.7	-	-	-	-	0.0	2.9	0.2	0.6
**49**	caryophylla-2(12),6(13)-dien-5*α*-ol	W, P2	1639	1634	0.3	0.4	1.0	0.5	0.4	1.4	0.6	0.3	0.6	0.7	0.6	2.4	1.3	1.7	-	1.1	3.0	2.4	0.6	0.8	0.0	3.0	1.0	0.8
**50**	caryophylla-2(12),6(13)-dien-5*β*-ol	W, P2	1639	1638	1.2	1.5	1.7	1.8	0.7	2.7	1.4	0.7	1.8	1.9	2.3	10.9	1.3	2.0	0.8	2.1	6.4	5.2	2.6	3.3	0.7	10.9	2.6	2.4
**51**	13-tetradecanolide	W, N, P1	1643	1642	0.4	0.4	-	9	0.2	0.2	0.2	0.2	0.2	0.2	0.1	0.6	0.1	0.1	0.1	0.1		0.1	0.1	0.3	0.0	0.6	0.2	0.1
**52**	*β*-eudesmol	W, P2	1649	1652	-	-	7.2	0.1	-	-	-	-	-	-	-	-	2.2	2.6	1.4	1.5	16.9	1.7	-	-	0.0	16.9	1.7	4.0
**53**	caryophylla-2(12),6-dien-5*α*-ol	W, P2	1643 ^e^	1660	0.1	0.1	-	0.2	0.3	0.3	0.1	0.1	0.2	0.3	0.2	0.7	-	-	-	-	-	-	0.2	0.3	0.0	0.7	0.2	0.2
**54**	sesquiterpene C_15_H_22_O	W, P1, P2	-	1668	0.3	0.8	0.1	0.5	0.9	0.8	-	-	0.5	0.3	-	-	-	-	-	-	-	-	-	-	0.0	0.9	0.2	0.3
**55**	caryophylla-2(12),6-dien-5*β*-ol	W, P2	1655 ^e^	1674	0.5	0.7	0.5	0.5	0.6	0.9	0.7	0.5	1.1	1.2	0.8	2.4	0.4	0.6	0.3	0.7	2.2	2.3	0.6	0.8	0.3	2.4	0.9	0.6
**56**	valeranone	W, N, P1	1674	1675	tr	0.1	-	-	-	0.1	0.1	-	0.4	0.1	-	0.4	-	-	-	-	-	0.2	tr	0.1	0.0	0.4	0.1	0.1
**57**	sesquiterpene C_15_H_22_O	W, P1, P2	-	1682	0.2	0.5	-	-	0.8	0.4	-	0.1	0.2	0.7	0.1	-	-	-	tr	-	-	-	0.2	0.3	0.0	0.8	0.2	0.2
**58**	germacra-4(15),5,10(14)-trien-1*α*-ol	W, P2	1685	1687	-	-	-	-	-	-	-	-	-	-	-	-	-	-	0.4	0.4	1.3	1.3	-	-	0.0	1.3	0.2	0.4
**59**	sesquiterpene C_15_H_26_O	W, P2	-	1695	0.3	0.4	0.4	0.4	0.1	0.6	0.1	0.3	0.3	0.5	0.6	1.2	0.1	0.2	-	-	-	-	-	-	0.0	1.2	0.3	0.3
**60**	14*α*-hydroxy humulene	W, P2	1714	1719	-	-	0.2	0.1	0.2	-	0.1	0.2	-	-	0.1	-	0.1	0.1	-	tr	1.4	1.3	0.1	0.2	0.0	1.4	0.2	0.4
**61**	chamazulene	W, N	1730	1728	-	-	-	tr	-	-	tr	0.1	-	0.1	-	-	tr	-	tr	0.1	0.1	-	-	tr	0.0	0.1	0.1	0.0
**62**	sesquiterpene C_15_H_24_O_2_	W, P2	-	1823	-	-	-	-	0.1	-	-	-	-	-	-	-	-	-	-	-	0.8	0.8	0.1	0.1	0.0	0.8	0.1	0.2
**63**	15-hexadecanolide	W, N, P1	1842 ^e^	1850	0.7	0.5	0.8	0.3	0.4	0.7	0.4	0.5	0.5	0.5	0.4	1.3	0.4	0.5	0.5	0.5	1.6	1.2	0.5	0.7	0.3	1.6	0.6	0.3
	monoterpene hydrocarbons				8.4	13.6	7.3	11.4	10.1	7.4	2.7	1.7	10.1	6.6	2.7	1.6	1.4	3.7	4.6	12.1	0.3	1.9	3.9	5.6	0.3	13.6	5.9	4.0
	oxygenated monoterpenes				79.7	70.1	76.5	79.2	73.9	73.3	89.0	90.4	72.1	76.3	83.1	57.2	89.4	83.3	73.4	70.6	43.2	59.5	81.7	75.0	43.2	90.4	74.9	11.4
	sesquiterpene hydrocarbons				0.8	1.1	0.6	0.7	0.6	0.9	0.9	0.6	1.8	1.9	1.0	4.9	0.8	0.9	1.0	2.0	2.4	2.7	2.4	1.7	0.6	4.9	1.5	1.1
	oxygenated sesquiterpenes				8.6	12.9	13.5	7.2	11.9	14.6	5.7	5.2	13.2	11.9	10.7	30.2	7.5	10.2	18.3	11.9	45.6	29.6	9.8	14.3	5.2	45.6	14.6	9.8
	others				1.1	0.9	0.8	0.3	0.5	0.9	0.6	0.7	0.7	0.7	0.5	1.9	0.5	0.6	0.6	0.6	1.6	1.3	0.6	0.9	0.3	1.9	0.8	0.4
	**total**				**98.6**	**98.6**	**98.7**	**98.9**	**97.0**	**97.0**	**98.9**	**98.6**	**97.9**	**97.4**	**98.0**	**95.8**	**99.5**	**98.8**	**97.9**	**97.3**	**93.2**	**95.0**	**98.5**	**97.5**	**93.2**	**99.5**	**97.6**	**1.5**

Note: tr = traces (<0.05%); >0.05% quoted to nearest 0.1%. ^a^ Compounds are listed in order of elution from an Elite-5 MS capillary column. ^b^ W = whole essential oil, N = eluted with pentane, P1 = eluted with pentane-5%Et_2_O, P2 = eluted with Et_2_O. ^c^ Retention indices from Adams 2007 [23] unless stated otherwise. ^d^ Retention indices determined on an Elite-5 MS column using a homologous series of n-alkanes under the same conditions as for samples. ^e^ According to [15].

These two lactones have previously been identified in the essential oils of some *Achillea* spp. [22] and are known to contribute to the distinct musky odor of the essential oils extracted from other plant species, such as *Angelica archangelica* L. (Apiaceae) [6,24].

With the aim of finding any possible correlation among detected compounds, a Pearson Correlation was carried out (Supplementary Table S1). Significant correlations were obtained for the most abundant compounds. Camphor (**21**) and 1,8-cineole (**11**) were positively correlated (0.591), while a negative correlation was found for these compounds and the two thujones detected, that is, *cis-* (**16**) and *trans-* (**17**) thujone, as reported in Table 3. These results agree with the literature data [25], which reported that camphane-type compounds (e.g., camphor, borneol, and bornyl acetate) and thujane-type compounds (such as *cis*- and *trans*-thujones) are the most commonly occurring monoterpenoids in *Achillea* essential oils. Further significative positive correlations were observed among *E*-caryophyllene (**35**) and all its oxygenate derivatives (Figure 1), that is, caryophyllene oxide (**42**) (0.767), caryophylla-2(12),6(13)-dien-5*β*-ol (**50**) (0.859), caryophylla-2(12),6(13)-dien-5-one (**41**) (0.877), caryophylla-2(12),6(13)-dien-5*α*-ol (**49**) (0.583), caryophylla-2(12),6-dien-5*β*-ol (**55**) (0.745), caryophylla-2(12),6-dien-5-one (**43**) (0.609) and caryophylla-2(12),6-dien-5*α*-ol (**53**) (0.547). This last result is indicative of an existing metabolic pathway oriented to the simultaneous synthesis of all these compounds, rather than of a possible transformation of caryophyllene in its oxygenated derivatives. An attempt was made with the aim of correlating the observed variability with the site of origin of investigated accessions, but the results were not consistent. The high variability observed in the composition of the analyzed samples can be explained first by the fact that the investigated accessions consist of cultivated plants that originated from natural populations, and the individual morphological, chemical and genetic variability among first-generation cultivated plants originating from natural populations is a well-known aspect [26,27]. Secondarily, the distilled plant material of each sample, which added up to 10 g dry weight, was randomly taken from a bulk sample collected in the experimental field, which originated from 30 plants per accession. Future research could be possibly be aimed at investigating the variation of the chemical composition among single plants.

## 3. Materials and Methods

### 3.1. Plant Material

Plants of *Achillea erba-rotta* subsp. *moschata* (Wulfen) Vacc. were cultivated in the same field trial as described in Fusani et al. [14]. The harvest of flowering tops, which is the commonly used plant part of the species, was performed at the second year of cultivation, on 14 June 2022, on ten cultivated accessions, that is, the seven already described in Fusani et al. [14] plus three more cultivated in the same conditions. Two representative samples of plant material (about 40–50 g fresh weight each) per accession were collected at the full blooming stage and successively dried in a thermostatic oven at 36 ± 1 °C for 48 h. Although collected in the same experimental trial and one the same date, the collected plant material samples differ from the one subject of the previous study [14]. Samples were authenticated by P. Fusani of the CREA Research Centre for Forestry and Wood, of Trento, Italy, where a voucher specimen of each investigated accession was conserved (AEM#202201-10).

### 3.2. Extraction of Essential Oil

Essential oils were obtained by the hydrodistillation of 10 g of dried plant material with 500 mL of water using a Clevenger type laboratory distiller (Universal, Milano Italy) with a 1 L capacity round-bottomed flask heated by a thermomantle for a one-hour period measured from the beginning of distillation, using 2 mL of diethyl-ether (Carlo Erba reagents, Cornaredo, Italy) in the burette to collect the essential oil. A single distillation was carried out *per* each harvested sample. The obtained essential oils were dried over anhydrous sodium sulfate and filtered, and the solvent was removed under a stream of N_2_. The essential oils were then weighed to calculate the recovery yield and stored in sealed vials with Teflon-lined caps at −20 °C until analysis.

### 3.3. Fractionation of the Essential Oil

A representative sample of the essential oil from *A. erba-rotta* subsp. *moschata* was obtained by mixing equal amounts (3 μL) of each of the essential oils obtained. A portion (2 mg) of a representative sample of the oil was placed onto a glass column (7 × 30 mm) of silica gel 60, 230–400 mesh (Merck, Darmstadt, Germany), preconditioned with fresh distilled pentane. The fraction containing hydrocarbons was eluted with 2 mL of pentane (fraction N). Successive elution with pentane-5% diethyl ether (2 mL) and diethyl ether (2 mL) afforded the fractions P1 and P2, respectively, containing the oxygenated compounds. Solvents were removed using a stream of N_2_, and fractions were analyzed by GC/FID and GC/MS.

### 3.4. GC/FID and GC/MS Analyses

GC/FID analyses were carried out using a Perkin Elmer Clarus 500 GC. For essential oils, an Elite-5MS capillary column (30 m × 0.32 mm, 0.32 µm film thickness) was used. Samples were injected (0.5 µL) in the “split” mode (1:30) with a column temperature program of 40 °C for 5 min, then increased to 280 °C at 4 °C min^−1^, and finally held at this temperature for 10 min. The injector and detector were set at 250 °C and 300 °C, respectively, and the carrier gas was He with a head pressure of 12.0 psi. GC/MS analyses were carried out using a Perkin Elmer Clarus 500 GC equipped (Perkin Elmer, Shelton, CT, USA) with a Clarus 500 mass spectrometer using an Elite-5MS capillary column (Perkin Elmer, Shelton, CT, USA, 30 m × 0.32 mm, 0.32 µm film thickness) and the same chromatographic conditions as for the GC/FID analyses. Mass spectra were acquired over the 40–500 amu range at 1 scan/sec, with an ionizing electron energy of 70 eV and the ion source at 200 °C. The transfer line was set at 300 °C, while the carrier gas was He at 1.0 mL min^−1^. The identification of all the compounds was performed by comparison with authentic reference standards as well as with published data and by a peak-matching library search [13,14,23,28]. The identification of the essential oil components was also performed by the determination of their retention indices (AI), which were calculated using a *n*-alkane series (C_6_–C_25_) under the same GC conditions as for the samples. The relative amount (%) of individual components was expressed as percent peak area relative to total peak area from the GC/FID analyses of the whole extracts.

### 3.5. Statistical Analysis

Data were elaborated by Pearson Correlation using the statistical software XLSTAT 2018.1 (Addinsoft, New York, NY, USA).

## 4. Conclusions

With 63 compounds detected and 59 identified, this was the most complete study on the characterization of the essential oil of *A. erba-rotta* subsp. *moschata* reported to date. In particular, 29 sesquiterpenes were quantitated, 25 of which were identified. Twenty-one compounds were identified for the first time in this species, with several oxygenated derivatives of caryophyllene among them. A great variability was found in the composition of the investigated *A. erba-rotta* subsp. *moschata* samples, and although it was not possible to group them according to the accession they originated from, a significative correlation was found among the most abundant compounds, supporting the hypothesis of the existence of different chemotypes within the species. This hypothesis assumes particular importance in the perspective of a possible breeding program, for which it could be worth considering selecting those plants characterized by a lower thujone content as the starting plant material. Further studies would be needed, however, in aiming to investigate the possible causes of variability of the essential oil composition within the species.

## Figures and Tables

**Figure 1:**
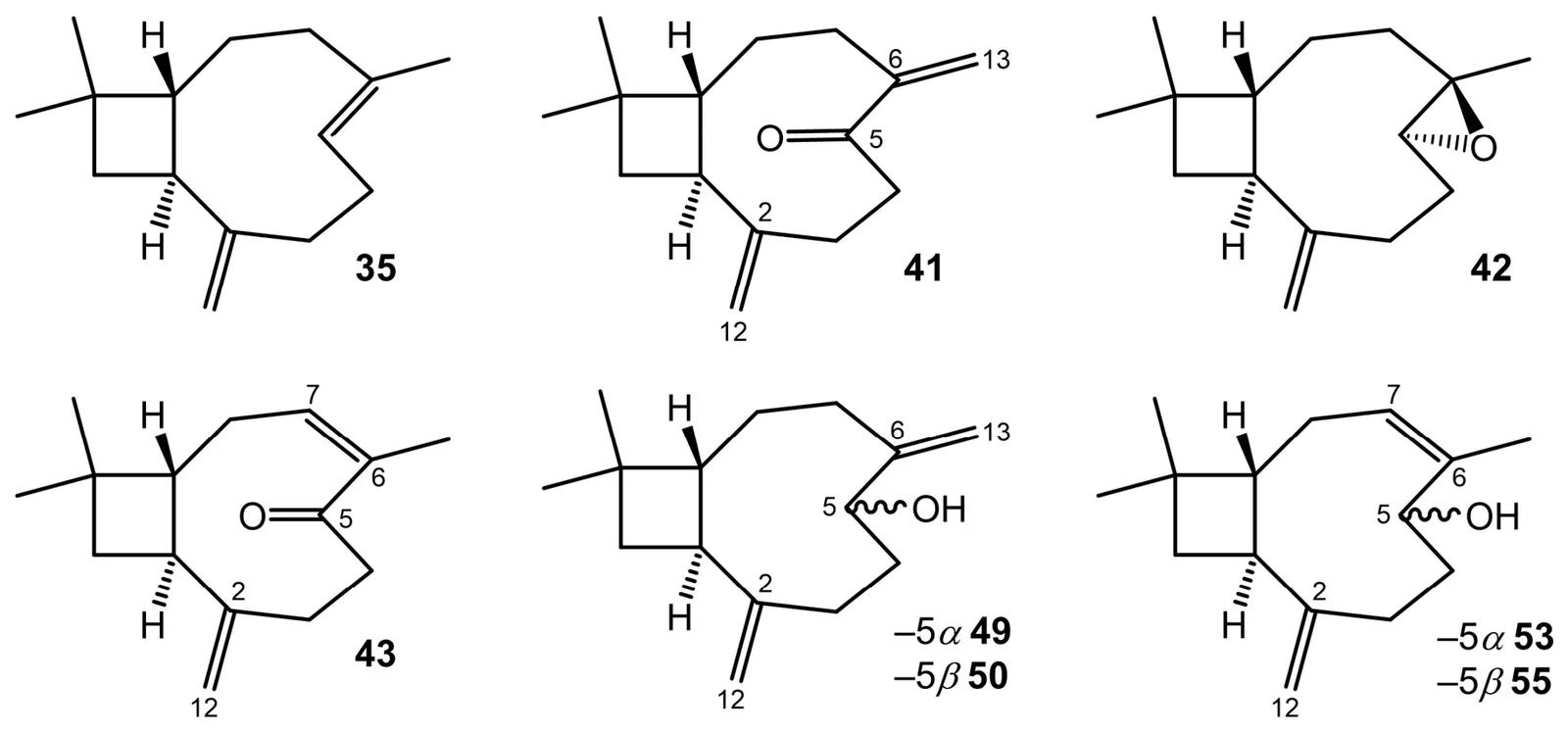
Chemical structure of caryophyllene (**35**) and its oxygenated derivatives identified in the essential oil of *A. erba-rotta* subsp. *moschata*.

**Table 1:** Essential oil yield (% dry weight) of the twenty investigated samples of *A. erba-rotta* subsp. *moschata* flowering tops.

Sample	Yield (%)	Sample	Yield (%)	Sample	Yield (%)	Sample	Yield (%)
1	0.42	6	0.39	11	0.61	16	0.52
2	0.35	7	0.39	12	0.34	17	0.28
3	0.35	8	0.43	13	0.52	18	0.33
4	0.36	9	0.41	14	0.39	19	0.38
5	0.38	10	0.40	15	0.55	20	0.32

**Table 3:** Correlation matrix between the contents of the most abundant volatiles identified in *A. erba-rotta* subsp. *moschata* analyzed samples ^(a)^.

Compounds	Camphor	*cis*-Thujone	*trans*-Thujone
*cis*-thujone	**−0.680 (0.001)**		
*trans*-thujone	**−0.640 (0.002)**	−0.227 (0.335)	
1,8-cineole	**0.591 (0.006)**	**−0.483 (0.031)**	**−0.468 (0.037)**

^(a)^ Pearson correlation; *p*-values indicated between parentheses. Bold typed values are different from zero at *p* < 0.05.

## Data Availability

The data are not publicly available. The data presented in this study are available on request from the corresponding author.

## Supplementary Materials

The following supporting information can be downloaded at: https://www.mdpi.com/article/10.3390/plants15203176/s1.
